# Understanding Suicide over the Life Course Using Data Science Tools within a Triangulation Framework

**DOI:** 10.20900/jpbs.20230003

**Published:** 2023-03-02

**Authors:** Lily Johns, Chuwen Zhong, Briana Mezuk

**Affiliations:** Center for Social Epidemiology and Population Health, Department of Epidemiology, University of Michigan School of Public Health, Ann Arbor, MI 48109, USA

**Keywords:** suicide, suicide prevention, life course framework, data science, triangulation

## Abstract

Suicide and suicidal behaviors are important global health concerns. Preventing suicide requires a nuanced understanding of the nature of suicide risk, both acutely during periods of crisis and broader variation over the lifespan. However, current knowledge of the sources of variation in suicide risk is limited due to methodological and conceptual challenges. New methodological approaches are needed to close the gap between research and clinical practice. This review describes the life course framework as a conceptual model for organizing the scientific study of suicide risk across in four major domains: social relationships, health, housing, and employment. In addition, this review discusses the utility of data science tools as a means of identifying novel, modifiable risk factors for suicide, and triangulation as an overarching approach to ensuring rigor in suicide research as means of addressing existing knowledge gaps and strengthening future research.

## INTRODUCTION

In 2020, approximately 46,000 people in the United States died by suicide, the 12th leading cause of death [1]. These deaths reflect a fraction of the population impacted by suicidality, however. In 2020, an additional 4.9% (12.2 million) US adults had serious thoughts of suicide in the past year, and 1.3% (3.2 million) made a suicide plan [2]. Besides the serious emotional and physical impacts on survivors and/or the social network of the deceased, suicide and suicide attempts also cause great economic costs. In 2019, the US spent nearly $490 billion on suicide and nonfatal self-harm [3]. In response to the increasing challenge of suicide, there has been significant renewed federal attention and efforts on suicide prevention. For example, in July 2022, the Federal Communications Commission adopted 9–8-8 as the official National Suicide and Crisis hotline [4]. Beyond interventions for acute crises, however, efforts at suicide prevention over the lifespan (e.g., addressing those factors that put people “at risk of risks”) [5] that contribute to suicide morbidity and mortality have received less attention.

Although it is well-established that a person’s risk of suicide varies by certain characteristics (e.g., demographics, genetics, geography, mental health history, sex, race, age, gender identity) [1,6–9], it is unclear what *modifiable* factors drive this variation in suicide risk. To date, research has been largely descriptive and focused on understanding the ‘who’ rather than the ‘why’ of suicide risk. This knowledge gap has been ascribed to methodological and conceptual challenges unique to the field. Most notably, Franklin and colleagues [10] conducted a meta-analysis of the last 50 years of research on suicide risk (and protective) factors and observed that, despite the growth in research and publications in this area, there has been little progress in determining the factors that predict suicide risk, and predictive ability has not improved over time. They found that current efforts at understanding suicide risk are largely limited to cross-sectional studies of well-established risk factors, and that these established risk factors are only modestly predictive of future suicidal behavior [10].

Franklin and colleagues also noted the disconnect between research and clinical practice in the field. Suicide risk is often conceptualized to be a complex combination of distal (e.g., history of depression, hopelessness, loneliness) and proximal factors (e.g., recent job loss or romantic breakup) that shape suicide risk [10–12]. However, most studies focus primarily on distal factors by studying the relationship between suicide risk and a certain risk factor (e.g., mental health history) over a long period of time [10], thereby under examining the impact of proximal factors on this relationship. This often translates to weak relevance for clinical practice because clinicians mainly assess suicidality based on acute risk [10]. Also, suicide death is a rare event and factors that are known to increase outcomes of suicidality are not clinically associated with an increased risk of such outcomes. For example, two individuals may have a similar risk for suicide based on such factors (e.g., both have a history of depression, both have access to a firearm), but only one exhibits suicidal behavior. Present efforts are inadequate to determine what features distinguish between these two scenarios and new methodological approaches are needed to close the gap between research and clinical practice. Here we outline suggestions for conceptual models, rigorous approaches, and novel analytic tools—namely, the life course framework, triangulation, and data science can address these gaps in knowledge and further our understanding of the nature of suicide risk, both acutely and over the lifespan.

## THE LIFE COURSE FRAMEWORK AS A CONCEPTUAL MODEL FOR UNDERSTANDING SUICIDE RISK

Previous suicide research and policy has focused on identifying “high-risk-groups” (e.g., men aged 50–69) or specific “high-risk times” (e.g., Monday) to inform suicide prevention efforts. However, this approach both assumes that people within a high-risk-group are homogeneous and does not address the underlying factors that make these groups and periods “risky” in the first place [13], both of which miss nuance within groups and can only inform prevention strategies in a limited way.

Drawing on Social Field Theory from developmental psychology, Chew and McCleary [13] questioned this assumption and applied the Life Course Framework as a means of understanding suicide risk. They argue that suicide risk is the product of *motivation* and *opportunity*. The life course framework emphasizes the importance of transitional periods where experiences in the life course may create diverging trajectories of risk, which in term shapes suicidal behaviors. Additionally, the opportunity of attempting suicide includes the possibility of surveillance (i.e., the probability of surviving a suicide attempt) and the accessibility of lethal means [13]. Importantly, motivation and opportunity are highly correlated; therefore, suicide research should consider them as a whole and situate people in the context of their life course, rather than viewing these as separate, discrete features.

**Figure 1.**
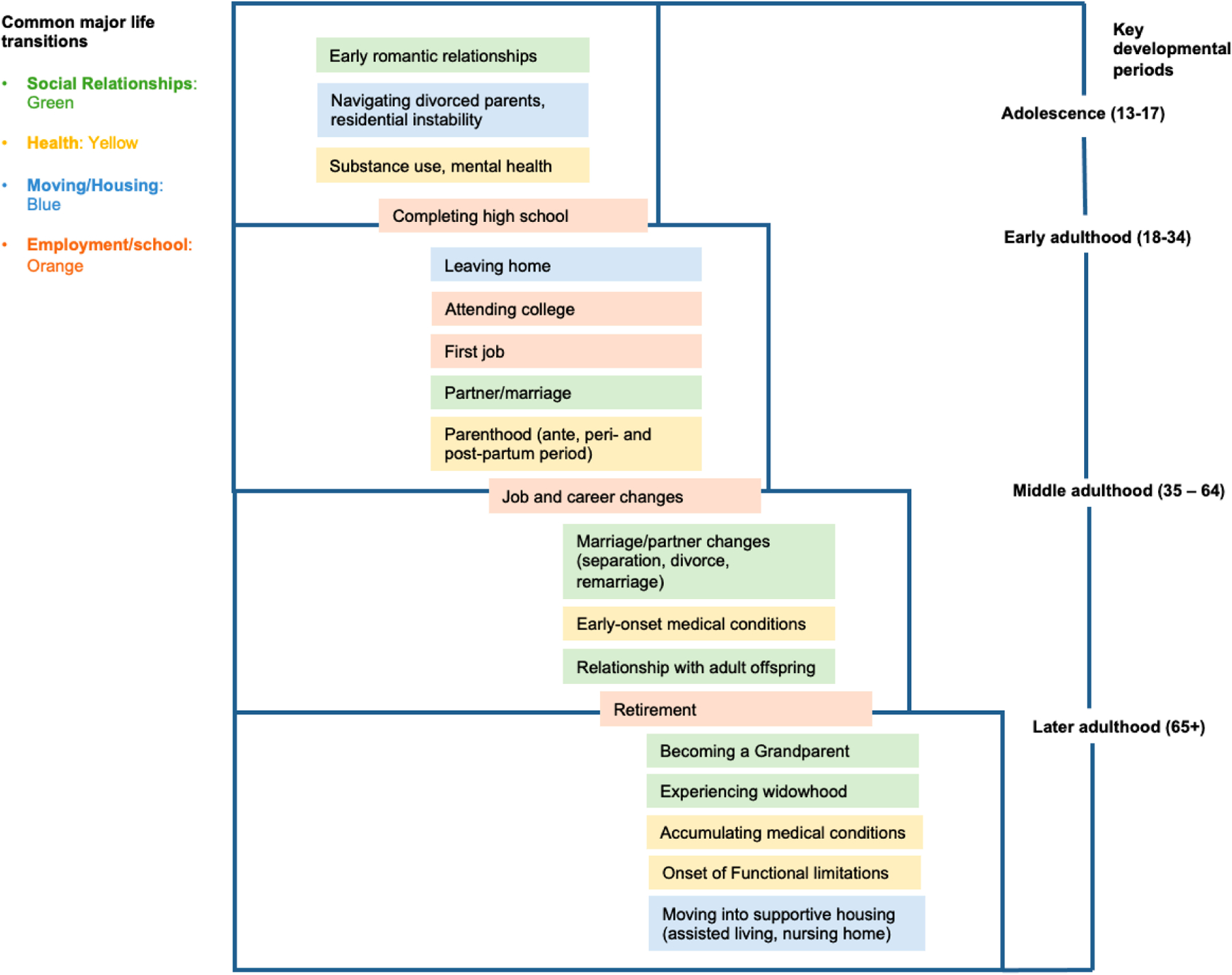
Key developmental periods and common life transitional events relevant to suicide risk.

Accordingly, the Life Course Framework proposes four periods, including adolescence, young adulthood, middle adulthood, and later adulthood, and four domains of life transitions, including social relationships, health and functioning, housing, and employment, in which to situate suicide risk (Figure 1). In this way, the Life Course/Social Field Framework unpacks what is a discrete, acute event (i.e., job loss) to investigate how the *timing* of that event, the characteristics of the *individual* experiencing that event, and the characteristics of the *setting* where that event occurred, relate to suicidal ideation, planning and attempt. Using the lens of the Life Course Framework, suicide prevention strategies may identify periods for assessing suicide risk and “points of engagement” for reducing suicide behaviors [14]. As evidence of the policy relevance of this framework, the US Surgeon General and the National Action Alliance for Suicide Prevention [15,16] have explicitly called for suicide prevention efforts to consider pathways and mechanisms that contribute to suicide risk from a lifespan perspective. Moreover, some states (e.g., Virginia) now explicitly refer to the life course framework in their plans for suicide prevention [17]. Table 1 summarizes examples of these points of engagement in the four domains, and they are described in detail below.

### Social Relationships

Social roles and connections have long been theorized as central determinants of suicide and suicidal risk behaviors (SRB, e.g., depressive symptoms, hopelessness). The importance of social connections is highlighted by the Interpersonal Theory of Suicide (ITS), a conceptual model that emphasizes thwarted belongingness (e.g., loneliness) and a lack of meaningful social roles, combined with perceived burdensomeness on others, as central to increasing suicide risk [18,19]. The ITS model calls attention to the importance of common transition events of social connection within the life course including a change in marital status, family conflict, and losing loved ones, all of which have been shown to relate to higher suicide risk. For example, a recent meta-analysis found that after adjusting for covariates, the relative risk of suicide in non-married individuals was estimated to be 92% higher compared to their married counterparts [20]. Beyond relationships, other social factors may be protective or risk factors for SRB over the life course (e.g., daily interactions, loneliness, living alone, spousal interactions, social networks, widowhood, caregiving) [21–23].

### Health

While it is recognized that mental health disorders, such as schizophrenia and depression, are associated with a higher risk of suicide [24,25], physical health issues are also correlated with SRB. Increasing long-term healthcare demands, lifestyle changes, and/or stigma towards having certain diagnoses, all of which are symbols of health declines that may cause stress, sleep problems, or a burdensome feeling, which may increase the suicide risk. Previous research has shown a link between certain health conditions and suicide, including those that are life-threatening (e.g., cancer) [26,27], as well as those that are chronic and involve functional impairment (e.g., diabetes and dementia) [28,29]. The mechanisms underlying the associations between diseases and SRB are multilayered and vary between diseases. For instance, diabetes is associated with higher suicide risk, possibly because of its established comorbidity with depression [28]. The association between diabetes and depression may be bi-directional, emphasizing the need to consider how this relationship may vary over the lifespan [30,31]. Beyond specific health conditions, some subclinical physiological states are also associated with elevated suicide risk; elevated levels of inflammatory biomarkers are associated with depressive symptoms and suicidal ideation [32,33]. In addition, emerging work has shown a link between placenta inflammation and post-partum depression and suicidality [34]. The pathways linking cancer and suicide risk appear to primarily reflect psychological coping factors [26]. Finally, specific periods of the patient’s journey are also associated with elevated suicide risk, particularly the few weeks immediately following discharge from a psychiatric hospitalization [35,36].

### Housing

Housing is a central social determinant of (mental) health [37] and housing issues can be disruptive, both on their own and as a correlate of precipitating changes (e.g., financial insecurity, changing jobs, functional decline) [38]. Furthermore, these effects vary throughout the different phases of the life course.

For younger adults, housing instability and mortgage delinquency are associated with anxiety, depression, and stress [39]. Foreclosure has been examined as a risk factor for SRB [40], and studies have shown that the US housing crisis in 2007 contributed to a rising rate of suicide deaths, emphasizing the role of macro factors in shaping suicide risk [41]. For older adults, house transitions are also associated with suicide risk, such as transitioning into residential long-term care (LTC; independent or assisted living, nursing home). There are about 16,000 nursing homes and about 31,000 assisted living facilities in the United States, and it is estimated that 52% of American adults aged 65 years and older will need some sort of LTC at some point in their lifetime [42]. Mezuk et al. [43] found that transitioning into residential LTC was related to risk for suicide, specifically that 2.2% of suicides among adults ≥55 were related to long-term care (i.e., assisted living facility, nursing home) in some manner, most commonly related to transitioning into or out of these settings. As the population ages, housing transitions such as these may be important points of intervention for mental health promotion.

### Employment

As emphasized by the recent US Surgeon General’s report on workplace mental health [44], work is an important setting for mental health promotion and suicide prevention. Several work characteristics and employment factors have been examined as risk factors for suicide. For instance, there is a well-established link between unemployment, including economic recessions and periods of economic uncertainty, and suicide risk [45–48]. Among those who are employed, several factors such as job insecurity, job strain, precarious employment (i.e., non-standard, part-time, or contingency work), and poor working conditions are associated with a range of poor mental health conditions [49–51]. In addition, employment is not a static feature, and most individuals go through multiple transitions (e.g., periods of unemployment, promotions, retirement) during their life course. As an example of how these work transitions may relate to suicide risk, a recent study [52] examined how retirement *expectations* (i.e., whether the transition was anticipated or not) related to depressive symptoms and passive suicidal ideation among older adults. They found that higher expectations of working past age 62 were inversely associated with depressive symptoms longitudinally and at baseline [52]. Other work has examined how “met” versus “unmet” expectations about retirement relate to mental health and shown that “unmet” expectations are associated with worse mental health [53].

The relevance of economic and employment context is also reflected by efforts in Japan and South Korea to address suicidality related to “overwork” [54–56]. These countries have among the highest rates of suicide in the world [57] and have undertaken specific policy changes related to work (e.g., mandates to reduce number of hours worked, programs to address burnout) [58–61]. Echoing these efforts, the US Surgeon General’s Call to Action for suicide prevention [62] also illustrates the need for policy interventions that strengthen economic supports at a population and community level (e.g., improved unemployment benefits, transfer payments) that can mitigate the impact of the risk associated with both the acute employment events and chronic employment and financial-related stressors.

## TRIANGULATION AS AN APPROACH TO INVESTIGATING SUICIDE RISK OVER THE LIFE COURSE

As mentioned above, the Life Course Framework aims to understand the complex variation in suicide risk over the lifespan by examining transitional periods or events. This effort requires a comprehensive epistemological approach to guide the testing of hypotheses. Specifically, *triangulation* is an overarching approach to research that can enhance rigor and reproducibility and provide a more comprehensive understanding of complex phenomena like SRB [63,64]. Drawing on the foundational underpinnings of mixed-methods research [65,66], as an approach to the research enterprise triangulation leverages multiple data sources, various analytic techniques, and diverse theoretical concepts to address a given research question This approach seeks separate the testing of hypothesized relationships from the specific analytic methods, which each have potential biases and limitations, used to test those relationships [63,64]. In the last two decades, triangulation has been widely applied to multiple disciplines, including sociology, nursing, and education [63,67,68]. As the drivers of SRB reflect a confluence of genetic, biological, psychological, social, and environmental factors, the field would likely benefit from adopting triangulation as an overarching epistemic approach to empirical research.

**Figure 2.**
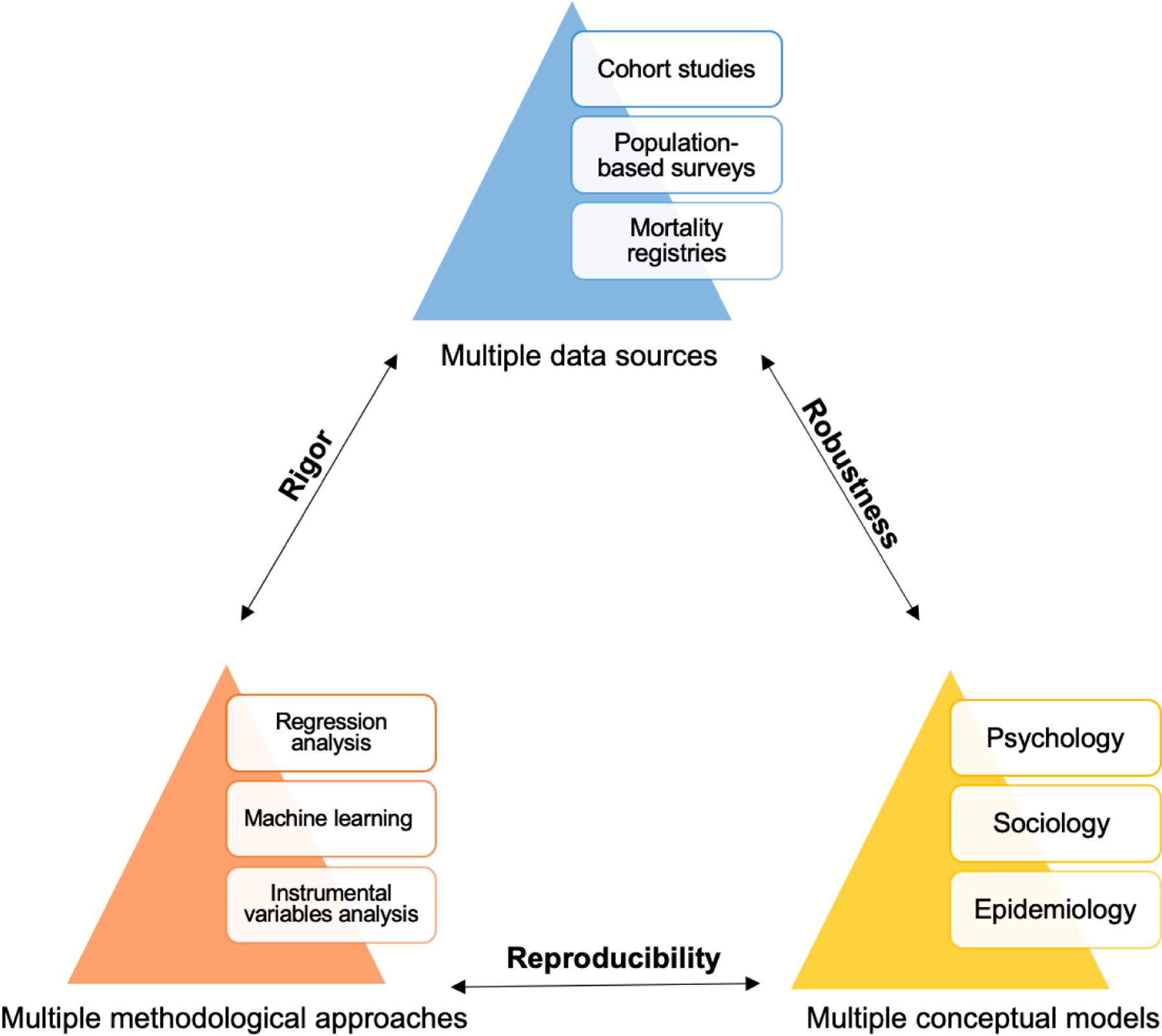
Conceptual model of triangulation for suicide research.

As shown in Figure 2, triangulation requires integrating (1) different conceptual models (e.g., sociological models that provide macro-level context factors and psychological models that provide individual-level factors), (2) multiple sources of data (e.g., longitudinal, population-based surveys that include with measures of SRBs over multiple time points as well as mortality registries that contain information on suicide deaths), and (3) multiple different analytic approaches (e.g., machine learning, regression analysis). Triangulation thus offers a means to redress some knowledge bottlenecks related to the study of SRB. For example, because suicide death is a rare event, the assumptions associated with regression-based statistical analyses (e.g., multivariate normality, homogeneity of variance) are tenuous which calls into question the validity of any observed associations. As a result, much of suicide research on risk and protective factors is descriptive, rather than analytic, in nature, with little innovation in developing new hypotheses or refining theories [10,11]. By comparing results across a variety of analytic approaches and datasets, triangulation enhances the value of all these data sources. Adopting this approach may allow the field to move beyond the “who” of suicide risk to a more nuanced and complete understanding of the “why”, which can more concretely inform prevention efforts. To achieve this, researchers need to both engage in team science [69], including community-engaged partnerships [70], and to embrace open science principles, including data and code sharing [71–73].

## DATA SCIENCE AS A TOOL FOR INVESTIGATING SUICIDE RISK OVER THE LIFE COURSE

One of the tools identified by Franklin and others as a means of addressing gaps in the field of suicide research are the models and algorithms (e.g., machine learning, artificial intelligence) encompassed by the field of “data science” [10,74,75]. Data science approaches are well-suited to analyzing large amounts of complex data, without assumptions regarding the distributions of variables. By not relying on generalized linear models, data science approaches can identify complex subgroups more efficiently than regression-based statistical models and can analyze information from thousands of variables simultaneously, therefore providing a more efficient means of identifying relationships that are not hypothesized a-priori [76]. As such, these tools are well-suited for identifying non-linear, complex relationships and for both testing hypotheses from existing theories and for identifying novel risk and protective factors for suicide that are not emphasized by current conceptual models.

Data science methods can primarily be applied to suicide research in two distinct ways: (1) as a tool of prediction and (2) as a tool of interpretation or description. For the former, data science can be used to analyze large amounts of complex data to create a model to predict suicide attempt or death in a given population and/or with a given risk factor. For example, previous research has used machine learning models to predict suicide death following psychiatric hospitalization among US soldiers [77] and to predict suicide attempts among adolescents in a longitudinal clinical sample [78]. Recent research [79] has also utilized machine learning to build a predictive model of suicide attempts model that uses identifies key risk factors associated with suicide attempt. While these are important advances, many studies of SRB that use data science/machine learning as a tool of prediction are often confirmatory and focus on established risk factors of SRB, rather than identifying novel risk factors. In addition, although predictive data science models of SRB do produce *statistically* significant classification accuracy [80], many only achieve positive predictive values of around 10% (i.e., only 1 in 10 of the future cases of SRB were identified by the algorithm) [79], which further demonstrates the challenge of weak clinical utility of extant suicide research. This illustrates the current limits of data science tools as a means of creating SRB prediction models.

Another use of data science methods is more exploratory, with the goal of identifying novel correlates of SRB that are not already established by existing conceptual models or prior empirical research. For example, data science tools can be used to describe analyze large amounts of unstructured textual data, whether from online forums, social media posts, or other sources. When combined with traditional analytic approaches (e.g., regression modeling), this application of data science to the field can help describe suicide risk as it relates to transitional periods or events across the lifespan. For example, previous research has used machine learning methods to study how life transitions intersect with suicide risk, including identifying suicide deaths related to transition into a long-term care facility [81] and examining suicide risk as it relates to driving cessation in older adults [82]. These studies have used natural language processing (NLP), a set of machine learning methods useful for identifying patterns in textual data, to examine thousands of textual elements from the National Violent Death Reporting System (NVDRS), a suicide mortality registry. They illustrate the ways in which data science tools can be used to generate hypotheses that can be tested in a variety of ways.

As data science tools become more widely adopted in in the field of suicide research, there are three areas that warrant consideration: (1) because these models are generated using the existing evidence base, they may reinforce existing biases or inequities due to insufficient data on racial and ethnic minorities, which is an emerging issue in the fields of health science [83,84]; (2) as there are not “gold standard” measures of suicide risk factors like depression, the ways in which these algorithms address (or fail to address) measurement error needs to be considered (e.g., while depression is an established risk factor for suicide, commonly used depression assessments used in public mental health research have only modest agreement with each other) [85]; and (3) strong predictive ability in a model or algorithm does not necessarily indicate a causal relationship [86]. That is, even if a certain risk factor (e.g., history of depression) is associated with a higher risk of suicide attempt, this information does not give substantive insight into why depression is associated with SRBs, or more importantly *how* future SRB can be prevented for people with a history of depression. In sum, data science methodologies are a promising and cost-effective tool for the field of SRB, although debate on how to best implement these tools is ongoing [87].

## CONCLUSIONS

Using conceptual models such as the Life Course Framework, integrated with triangulation as an overarching approach to scientific research and broader use of data science tools, has the potential to increase our understanding of suicide over the life course. Such efforts can help refine current theoretical models of suicide risk and inform innovative strategies for prevention. Current suicide prevention strategies are a reflection of the status and stagnation of the field [10]. Most approaches focused on proximal ‘warning signs’ connected to suicidal behaviors [88–90], focused on certain high-risk groups (e.g., military personnel and veterans, LGBTQ youth, college students) [91–93], or emphasized harm reduction strategies such as limiting access to firearms [94]. These efforts, while needed, are piecemeal and incomplete. Understanding how suicide risk emerges and abates over the life course, across different contexts, for different populations, requires interdisciplinary collaborations with both researchers and affected communities and stakeholders.

## Figures and Tables

**Table 1.** Examples of transition events across four domains over the life course.

Domain	Exemplar Transition Events
**Social relationship**	Changes in social network (e.g., change in marital status, death of a loved one) Challenges in existing social relationships (e.g., family conflict, negative daily interactions) Other social factors in daily life (e.g., feelings of loneliness, caregiving burden)
**Health**	Chronic disease (e.g., dementia, diabetes, chronic pain) or certain physiological conditions (e.g., inflammation) Recent discharge from a psychiatric care facility Recent diagnosis (e.g., cancer)
**Housing**	Moving into a residential long-term care facility Housing insecurity, eviction, or mortgage delinquency
**Work**	Retirement and retiring earlier than expected or expectations surrounding retirement Unemployment following economic recession or recent job loss Experiencing problems in the workplace, including workplace discrimination, job strain, or insecure employment (i.e., non-standard or contingency work)

## Data Availability

Data sharing not applicable to this article as no datasets were generated or analyzed during the current study.

## ABBREVIATIONS

CDC: Centers for Disease Control
LGBTQ: lesbian, gay, bisexual, transgender, queer or questioning
SRB: suicidal risk behavior
ITS: interpersonal theory of suicide
LTC: long-term care
NLP: natural language processing
NVDRS: National Violent Death Reporting System
